# A Finite Element Investigation into the Cohesive Properties of Glass-Fiber-Reinforced Polymers with Nanostructured Interphases

**DOI:** 10.3390/nano11102487

**Published:** 2021-09-24

**Authors:** Mohammad J. Ghasemi Parizi, Hossein Shahverdi, Ehsan Pipelzadeh, Andreu Cabot, Pablo Guardia

**Affiliations:** 1 Aerospace Engineering Department and Center of Excellence in Computational Aerospace, AmirKabir University of Technology (Tehran Polytechnic), Tehran 159163-4311, Iran; m.qasemi@aut.ac.ir; 2 Catalonia Institute for Energy Research—IREC, 08930 Sant Adrià de Besòs, Spain; ehsanpipelzadeh@hotmail.com (E.P.); acabot@irec.cat (A.C.); 3 Chemical Engineering Department, University of Queensland, Brisbane 4072, Australia; 4 ICREA, Pg. Lluís Companys 23, 08010 Barcelona, Spain; 5 Institute of Materials Science of Barcelona (ICMAB-CSIC), Campus UAB, 08193 Bellaterra, Spain

**Keywords:** glass-fiber-reinforced polymers, interfacial shear stress (IFSS), finite element, pull-out test, interphase, silica nanocrystals, graphene oxide nanosheets

## Abstract

Glass-fiber-reinforced polymer (GFRP) composites represent one of the most exploited composites due to their outstanding mechanical properties, light weight and ease of manufacture. However, one of the main limitations of GFRP composites is their weak inter-laminar properties. This leads to resin delamination and loss of mechanical properties. Here, a model based on finite element analysis (FEA) is introduced to predict the collective advantage that a GF surface modification has on the inter-laminar properties in GFRP composites. The developed model is validated with experimental pull-out tests performed on different samples. As such, modifications were introduced using different surface coatings. Interfacial shear stress (IFSS) for each sample as a function of the GF to polymer interphase was evaluated. Adhesion energy was found by assimilating the collected data into the model. The FE model reported here is a time-efficient and low-cost tool for the precise design of novel filler interphases in GFRP composites. This enables the further development of novel composites addressing delamination issues and the extension of their use in novel applications.

## 1. Introduction

Glass fibers (GFs) are an ideal and affordable primary reinforcement material, providing high strength and mechanical stability to polymer [1,2], concrete [3] and orthodontic [4] composites. However, one of the main limitations of GF-reinforced composites are their weak inter-laminar properties, including delamination resistance and crack propagation at the GF to polymer interphase [5]. These failures take place as a consequence of the poor mechanical stability of the interphase due to a low bonding energy between the fiber and the polymer matrix [6]. This issue has been partially addressed by developing different surface modification strategies on GFs [7,8].

Nowadays, nano- and microengineering methods are widely exploited to modify material properties providing different structures or textures [9,10]. In this regard, nanomaterials such as silica nanoparticles or graphene nanosheets have garnered much interest in the last decade as potential fillers to combine with fibers in polymer matrices [11]. Nonetheless, an effective interphase design to modulate the filler to matrix interaction, as well as a proper mechanical characterization of it, remains a challenge to be addressed [12].

Generally, resin–fiber interface modification using various structures is proposed as one of the most appealing solutions. The material is usually a mesoporous structure produced on the GF surface. Some researchers have already demonstrated this concept by growing different porous structures on top of GF with remarkable enhancement of the mechanical properties of the polymer composites [8]. In the past, the improvement of these mechanical properties was directly related to the morphology of the surface coating grown on the GFs. Although the characterization of the properties of the fiber to matrix interphase and interpenetrating region was not possible, sliding contact tests provided a qualitative study of the adhesion energies for each coating. Nonetheless, these experiments were carried out on top of modified glass substrates and not on GFs, which does not fit with the real operating conditions.

A proper characterization of the interphase is crucial to evaluate the adhesion energy and further the enhancement of the mechanical properties. Currently, this is achieved by micromechanical tests such as single and bundled fiber pull-out tests, which facilitate evaluation of the interfacial shear stress (IFSS) [13,14]. It is worth emphasizing that reported analyses of failure regions on laminated composites have shown fiber bundles pulled out from the specimen [15]. Thus, the failure mechanism in composites can be fairly characterized by a fiber bundle pull-out test. In addition, the IFSS can be directly correlated with adhesion energy between the fiber and matrix, thus providing a characterization of the fiber to matrix interphase in GF-reinforced composites [16].

As discussed above, single and bundled fiber pull-out experiments provide the utmost proof for the mechanical characterization of a composite, yet this is a costly and time-consuming process. In this regard, analytical tools and simulation models are useful to validate experiments while providing additional information when designing a composite [17,18,19]. For instance, molecular dynamics simulations have been recently exploited to study interfacial binding energy and failure behavior of GF/PEEK composites [20]. Similarly, micromechanics-based constitutive models have been able to predict the viscoelastic behaviors of particle-reinforced composites [21]. Those models, although being quite accurate, require advanced modelling tools and long calculation times. In the last decade, finite element analysis (FEA) has also been used to investigate interphase properties on GFRP composites [22,23]. Since the failure of GFRP takes place without any apparent sign or little evidence of material deformation, the understanding of debonding time with respect to the critical design parameters is important. Recently, Heyer and coworkers demonstrated the tremendous potential of FEA by developing an analytical model to predict the fatigue life of GFRP composites [24].

Compared to other analytical tools, FEA is able to provide reasonably accurate results in adequate calculation times [25]. For example, Lin and coworkers successfully simulated fiber pull-out mechanisms and the effects in composites by exploiting FEA, while Wei et al. were able to evaluate shear stress distribution across the interphase for the single pull-out test [26,27]. In both works, cohesive zone modeling (CZM) was exploited to evaluate the damage at the interphase and to evaluate fracture toughness; moreover, both studies employed the cohesive damage model to simulate damage initiation in the interphase [28].

Despite the recent advances in FEA, its reliability and accuracy does depend on the size and type of the mesh, boundary conditions and material models. Hence, it is difficult to provide an accurate yet simple model. In particular, for GFRP, describing the properties of the fiber to matrix interphase as a function of the surface coating in a simple model is a rather challenging task [22]. On the other hand, a more accurate model leads to higher computational times. This calls for the development of new models (based on parameters such as adhesion, effective polymer to filler contact area, and interphase structure or texture) that are able to extrapolate the influence of nanostructured coatings over the mechanical properties of composites.

In the present work, FEA and pull-out experimental data were combined in order to establish and validate a micromechanical model that provides the mechanical properties of a GF-polymer interphase. Here, in a simplified assumption, GFs were assumed to have homogenous regions, and their axial symmetry allowed for a simple 2D model. The GF to polymer matrix interphase region was simulated as a cohesive zone consisting of cohesive elements with perfect bonding conditions, parametrized by a damage parameter. FEA was combined with experimental results carried out on four different samples that had different types of porous structures grown on top of the GF’s surface. Here, silica microporous (SimGF), silica mesoporous (SiMGF), silica gel (SiGGF) and graphene gel (GGF) films were prepared and examined. For all the GFRP composites, fiber bundle pull-out tests were carried out to measure the cohesive surface energy through the evaluation of the maximum pull-out load. These data were further exploited for FEA by applying a cyclic corrective approach. The validation of FEA against experimental data was carried out to investigate the GF to polymer matrix interphase properties of GFRP composites as a function of GF surface morphology. In this study, structure properties such as pore size and specific surface area were correlated with the GF to matrix adhesion energy. The IFSS was directly compared to the specific surface area, thus providing key information on the surface coating of fillers in reinforced polymer composites.

## 2. Experimental Section

### 2.1. Sample Preparation

Up to four different GFRP composites were prepared using GFs with up to four different surface coatings. Silica microporous (SimGF), silica mesoporous (SiMGF), silica gel (SiGGF) and graphene gel (GGF) films were developed. Details for the synthesis along with the morphological characterization of the interfaces are described in the Supplementary Material (Figure S1).

### 2.2. Fiber Bundle Pull-Out Test

Fiber bundle pull-out experiments were performed in a home-made set-up using 5 cm-long GF bundles. Samples were prepared by growing an epoxy drop on top of a surface-modified GF bundle; specifically, a ca. 10 μL epoxy drop was placed at the bottom of a vertically oriented plastic mold made of 2 inverse cones to form a 1400 µm spherical cap (Figure S2a). A 5 cm GF bundle was then passed through the cones and partially immersed in the epoxy spherical cap. The sample was left to dry for 24 h at room temperature. Samples were characterized by a 100 µm radius GF bundle embedded ca. 700 µm into a 1400 µm epoxy spherical cap (hereafter epoxy cap). For the test, one of the ends of the bundle was clamped to a load cell (Degraw 5 kg Load Cell). The epoxy cap was then placed inside a steel holder attached to a software-controlled (Pewin32 Pro2) XY translation stage (PI miCos linear stages PLS-85 with 10 mm range in both X and Y with RS422 encoders) (Figure S2b–e). Before starting the experiment, the stage was slowly moved in order to tighten the GF bundle so that it remained parallel with respect to the table. Then, the stage was moved at a speed of 1mm/min and the displacement versus load curve was acquired. Each point was calculated from the mean value of at least 20 measurements (Figure S3). Before each pull-out test, the load cell was calibrated in order to avoid the hysteresis effect during the experiments.

## 3. FE Model

**Figure 1:**
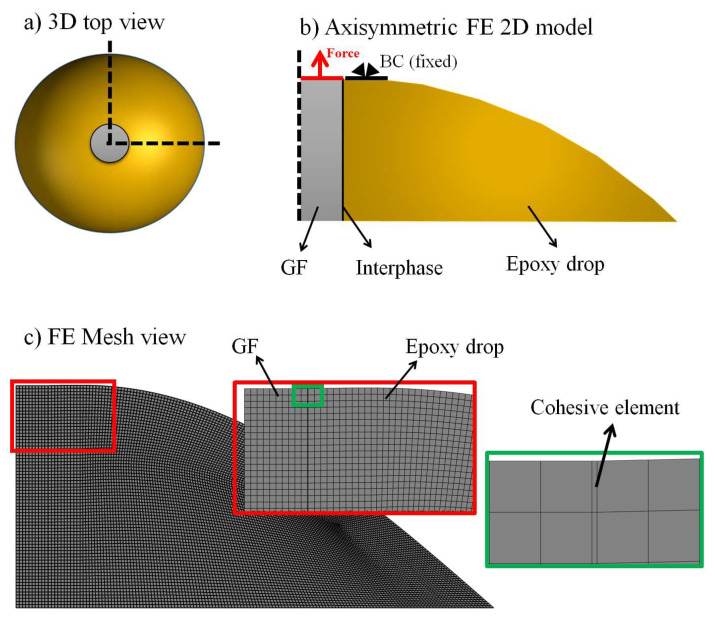
3D sketch of a GF bundle–epoxy cap (**a**), and the 2D sketch of the FE model for pull-out test simulations (**a**,**b**). Gray areas represent the GF bundle while orange areas represent the epoxy cap. The axial symmetry of the 3D GF bundle–epoxy model allows for FE simulations using a simplified axisymmetric 2D model. The GF–epoxy interphase was simulated using cohesive elements at the interphase (green frame in panel (**c**)).

The numerical model is validated using an FE model. The FE model is intended to mimic the pull-out test for the GF-bundle-embedded epoxy. In this case, GFRP composite models are symmetrical along their fiber axis, and thus an FEA 2D axisymmetric model using ABAQUS is feasible. Here, to develop the FE model, the object was divided into 3 regions, namely, (I) the GF bundle (here, for the sake of simplicity, the GF bundle was simulated as one homogenous fiber), (II) the matrix to fiber interphase and (III) the epoxy drop (or polymer matrix) (see Figure 1). The experimental parameters were: GF radius (100 µm), GF bundle embedded length (ca. 700 µm) and epoxy drop radius (1400 µm). The thickness of the interphase was assumed to be 1 µm. For the simulations, the properties were described as: E = 0.992 GPa and υ = 0.3 for the epoxy and E = 75 GPa and υ = 0.2 for the GF bundle as reported elsewhere [17]. CAX4 elements (4-node bilinear axisymmetric plane stress elements) were applied to reproduce the GF bundle. To simulate the cohesive behavior of the composite during the loading, the GF bundle–epoxy interphase was modelled using COHAX4 elements (4-node axisymmetric cohesive elements) with 70 linear quadrilateral elements. In our study, we concluded that 70 elements converge best and that more elements are proven to increase computational time without any meaningful contribution to the results (Figure 1). The model was meshed in a way that meant the displacement continuity must be satisfied under the border of two elements (conformity condition of elements).

The epoxy was modelled using the same elements employed for the GF bundle. The fiber and matrix bundle had 9945 linear quadrilateral elements (Figure 1). The interphase region was treated as a cohesive junction where the Mode II fracture (N/mm) was calculated and iterated to converge. The failure model was based on a CZM [29,30] with cohesive elements monitored by the scalar stiffness degradation (SDEG) parameter, which ranges from 0.0 to 1.0. The SDEG parameter stands for the damage evolution, and the failure criterion is satisfied when SDEG is 1.0. Note that this accounts for the total damage caused to the cohesive element and the subsequent crack formation [31]. Interfacial strength and penalty stiffness values for the interphase (5 MPa and 10^9^ N/mm^3^_,_ respectively) were extracted from the literature to the maximum pull-out load measured in the pull-out test (Figures S2–S4) [23,31,32,33,34]. Employing a systematic approach, the simulation outputs were compared against the experimental data for all surface coatings. This approach is thought to be useful in developing future modifications in material and composite engineering.

## 4. Result and Discussion

Previous studies showed that the filler-to-matrix interphase dominates the load transfer efficiency for laminate composites [35]. In this study, FEA and experiments for pull-out tests were combined to understand the influence that interphase properties have over the mechanical behavior of GFRP composites. In particular, we examined different types of interphases on GF with different porosities, specific surface areas and chemical affinities (see Figure S1).

**Figure 2:**
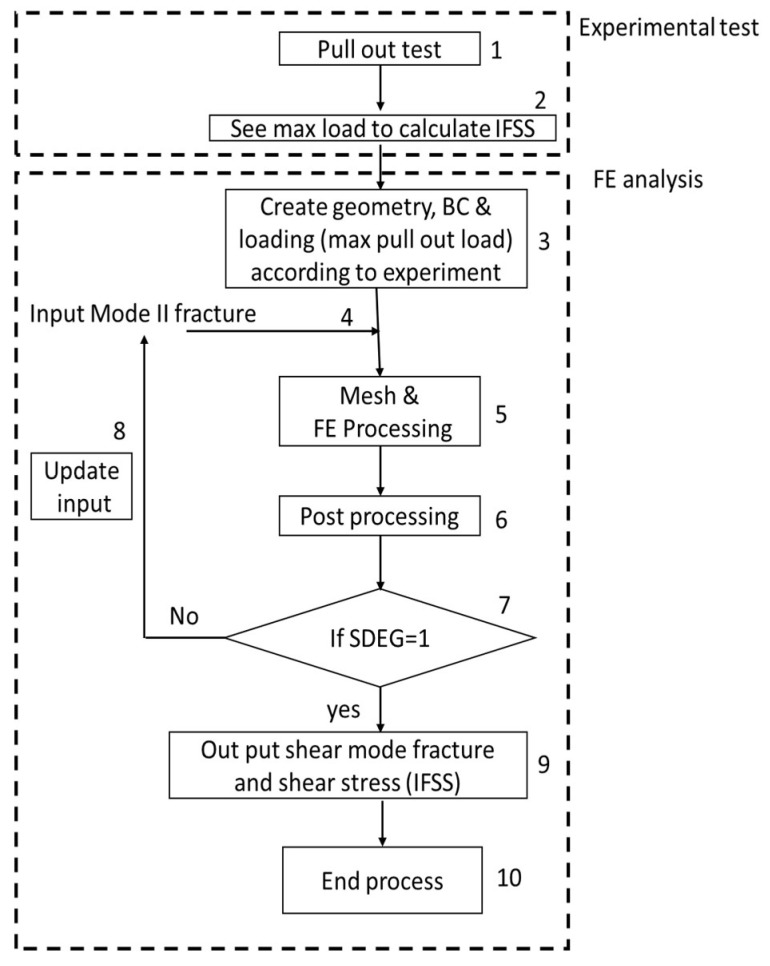
Data flow for experimental and computational pull-out test.

**Figure 3:**
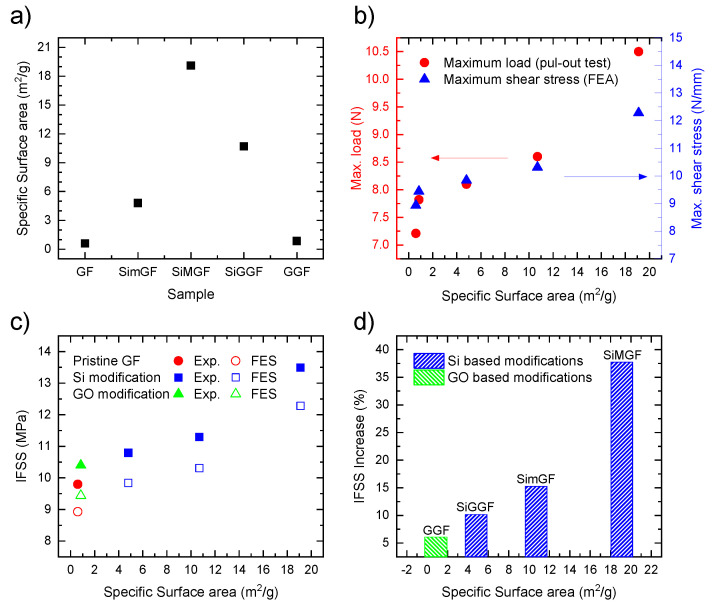
(**a**) Summary of the specific surface area values calculated using the BET model for all samples; (**b**) Maximum load (red circles, left y axis) and maximum shear stress (blue triangles, right y axis) as a function of the specific surface area obtained from the pull-out test and FEA, respectively; (**c**) IFSS values as a function of the specific surface area for composites prepared with GFs (GF, red circles), silica-modified GFs (SimGF, SiMGF and SiGGF, blue squares) and GO-modified (GGF, green triangles). Full data points represent experimental IFSS values, while empty data points represent calculated IFSS (see Table 1); (**d**) Improvement (in %) over the IFSS value as a function of the specific surface area for silica (blue bars) and GO (green bars) modified GFs.

As illustrated in Figure 2, our approach was divided into two main blocks. First, experimental pull-out tests were carried out to calculate the maximum pull-out load (Figure 2, entries 1 and 2, and Figure S3). As reported elsewhere [16], assuming a homogeneous stress distribution along the interface, the IFSS can be calculated as [36]:

(1) $$\mathrm{IFSS}=\frac{F_{\max}}{A}$$

where F_max_ is maximum pull-out force and A is the embedded area of the bundled GFs in the epoxy drop. Experimental pull-out tests were performed for all modified GFs as well as for pristine GFs. The IFSS was calculated using an applied maximum force, evaluated as the average value of 20 specimens (Figure S3), and a surface area of 0.44 mm^2^ (Table 1 and Figure 3).

**Figure 4:**
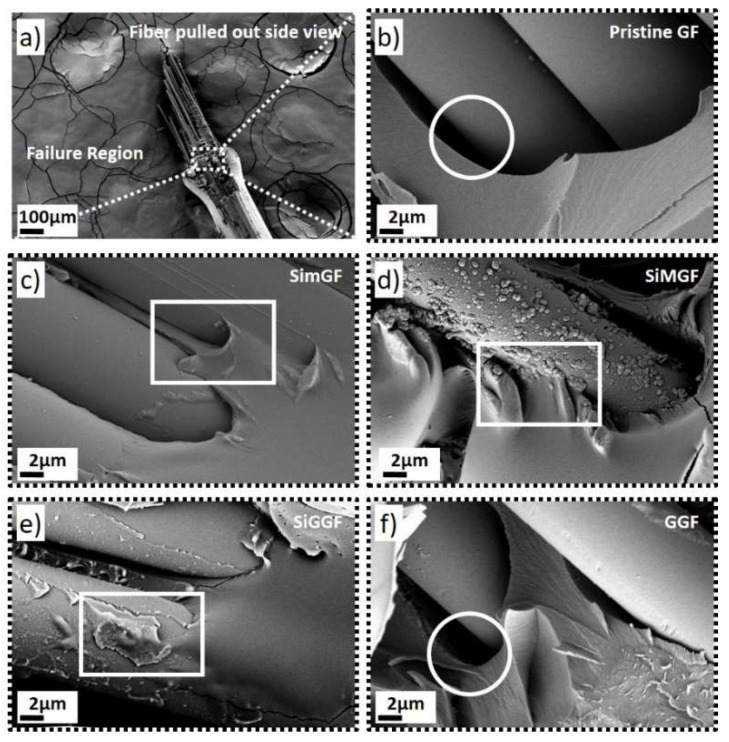
(**a**) Low magnification SEM image of a failure region (dashed rectangle) after pull-out test. (**b**–**f**) High magnification SEM images of failure regions for pristine GF, SimGF, SiMGF, SiGGF and GGF samples. Debonding and physical interlocking areas are highlighted with circles and rectangles, respectively.

After evaluating the experimental applied maximum force (or maximum pull-out loads) for each sample, these values were introduced into the FE model along with the boundary conditions (entries 3 and 4 in Figure 2). In order to simulate the GF-to-epoxy interphase, FE simulations were carried out using cohesive elements. As described above, the design criterion for the damage at the interphases was simulated by defining a damage initiation onset (based on strength criterion) and a damage parameter. For ABAQUS code, the damage state of single elements is monitored by the SDEG parameter [37]. Note that the debonding mechanism is assumed to take place at both sides of the interphase (Figure 4a). Finally, the boundary conditions were set as follows: (i) elements cannot be entirely damaged even if the initiation criterion is satisfied, and (ii) crack propagation implies complete damage to the elements.

During the FEA, the interphase properties (i.e., Mode II fracture) were updated at every cycle (entry 8, Figure 2) until the model converged (SDEG = 1). In other words, when the condition was satisfied, the damage at the interface was considered and the Mode II fracture and IFSS were delivered as outputs (entry 9, Figure 2). The results from the FEA were compared with the experimental data (Table 1 and Figure 3c). Note that the comparison between the experimental and calculated IFSS values validates the model. Along with these data, a summary of the surface specification and mechanical properties is presented in Table 1.

As reported in Table 1, all composites produced with modified GF demonstrate improvements in the experimental IFSS compared to those produced with pristine GF from 9.8 up to 13.6 MPa, which represents a 38% improvement (Table 1 and Figure 3d). The same trend was observed for FEA with values in sound agreement with the experimental data. For instance, 9.8 versus 8.9 MPa was recorded for pristine GF (for the pull-out test and FEA, respectively) and 10.8 versus 9.8 MPa for SimGF (for the pull-out test and FEA, respectively). The highest IFSS and maximum shear stress values were measured for SiMGF, which shows the highest specific surface area. This trend allowed us to directly correlate an increase in IFSS to the formation of the porous structure (Figure S1). However, in this assumption, the morphology of the porous structure is not considered. Indeed, this could play a key role in enhancing interlocking by the penetration of epoxy inside interconnected pores.

In order to gain further insight into this trend, we closely compared the data measured for SimGF and SiMGF. SiMGF was produced by growing mesoporous silica on the GF surface using wet chemistry (Figure S1). These types of structures are supposed to provide a higher specific surface area compared to the nanochannel surface assembly observed for SimGF. In particular, the specific surface area increased from 4.8 to 19.1 m^2^/g (for SimGF and SiMGF, respectively), which represents a ca. 400% increase. This significant improvement over the fiber-specific surface area definitively contributed to a positive enhancement of the adhesion between the GFs and epoxy. Nonetheless, the improvement in the experimental IFSS for SiMGF compared to SimGF was of ca. 25% (10.8 versus 13.5 MPa for SimGF and SiMGF, respectively). FEA provides similar IFSS values for SimGF and SiMGF (9.8 and 12.3 MPa, respectively) with the same deviation (25%).

Following the above reasoning, a gel structure was grown on the GF by means of a sol–gel approach (SiGGF, Figure S1). Gels provide an interconnected porous network, thus increasing the specific surface area while boosting interlocking between the GF and the epoxy. In this work, GFs modified with a silica gel structure on top (SiGGF) were characterized for lower specific surface areas compared to SiMGF (10.7 versus 19.1 m^2^/g for SiGGF and SiMGF, respectively). This could be ascribed partially to the collapse of pores during the drying process [38]. As for the IFSS, SiGGF samples showed slightly higher IFSS values compared to SimGF but lower values compared to SiMGF. This is in agreement with the hypothesis of the close entanglement between specific surface area and IFSS, but with some deviation: the IFSS values for SimGF and SiGGF were 10.8 and 11.4 MPa, respectively, while the specific surface areas were 4.8 and 10.7 m^2^/g, respectively. This is a 108% improvement to specific surface area and a 5.5% improvement of IFSS. The deviation between the improvements to the specific surface area (108%) compared to the IFSS (5.5%) could be attributed to a poor adhesion of the coating film (gel) grown on top of the GF surface. Finally, GFs were modified with a graphene gel coating (GGF, Figure S1). For this sample, the lowest IFSS and specific surface area were measured and compared to the other modified GF samples (0.8 m^2^/g and 10.4 MPa, respectively).

Experimental data were compared to values calculated from FEA. For all samples, experimental and calculated IFSS values were in close agreement with a deviation of less than 11%. This highlights the good correlation between the developed FE model and pull-out experiments. The specific surface area and IFSS values observed could be ordered as follows: BETSiMGF > BETSiGGF> BETGGF ≈ BETSimGF and IFSSSiMGF > IFSSSiGGF > IFSSGGF≈ IFSSSimGF. Furthermore, the relation between the IFSS and specific surface area followed a quasi-linear trend (Figure 3). Finally, the Mode II fracture increased as a function of the specific surface area, showing a maximum value of 10.5 N/mm for SiMGF samples. The increase in the Mode II fracture was of ca. 45%. For instance, composites produced with SiMGF showed a Mode II fracture of 10.5 N/mm compared to 7.2 N/mm when produced with pristine GF. From the above observations, we conclude that porous surface coatings have a positive impact over the mechanical properties at the GF-to-epoxy interphase of GFRP composites. In particular, the porous structure contributes to a more homogenous load transfer, better physical interlocking and a decrease over the stress accumulation at the interface.

The interfacial adhesion between GF bundles and the epoxy was qualitatively evaluated by SEM (Figure 4, Figures S5 and S6). Figure 4a shows a representative low-magnification SEM image of a damage area after a pull-out test (dashed frame). A higher magnification of the failure regions for each sample provides insight into the failure mechanism at the interphase as a function of the surface modification (Figure 4b–e and Figure S5). For instance, large, detached areas were observed at the failure region for samples produced with pristine GF and GGF (circles in Figure 4b,f) suggesting an inadequate GF–epoxy contact. This results in a non-uniform load distribution and, hence, a stress concentration, which promotes surface microcracks. This is potentially responsible for a decrease in the elastic properties at the interphase, and thus, in firm agreement with the poor mechanical properties calculated for GF and GGF composites (Table 1). For SimGF, SiMGF and SiGGF composites, a different failure region was observed with areas showing that the epoxy matrix was well-embedded into the GF surface modification (squares in Figure 4c–e). This enhancement of the contact between GF and epoxy is likely attributed to an increase in the surface area and an enhancement of the physical interlocking for the GF coating. As such, load is homogenously distributed along the interphase, thereby reducing stress concentration. Moreover, the nanostructured coatings at the interphase could also limit crack propagation, acting as an intermediate multi-structure sandwiched between the GF and the epoxy drop. This is in firm agreement with the enhancement of the mechanical properties (Table 1).

## 5. Conclusions

In this work, FEA was combined with an experimental pull-out test to study the mechanical properties at the filler-to-matrix interphase on GFRP composites. In particular, we aimed to study the influence that a filler’s surface coating has on the mechanical properties of the composites. On the one hand, porous silica- (SimGF, SiGMF and SiGGF) and graphene-based (GGF) surface coatings were grown on top of GF. An epoxy drop was then deposited onto the modified and pristine GF bundles to carry out the pull-out test. The IFSS values were calculated by extracting the maximum force from the force-versus-displacement curves. SEM images of the damaged areas after the pull-out test were taken to study the delamination mechanism. All data were compared with the specific surface area measured for each sample. On the other hand, a 2D axisymmetric model was developed to run FEA on the pull-out test. The model addressed the GF-to-epoxy interphase properties by using cohesive elements with perfect bonding conditions. To calculate the cohesive properties at the interphase, the maximum load measured by the pull-out test was introduced in an iteration process that ran until the SDEG values converged to one.

Experimental tests and FE simulations revealed that IFSS values increased as a function of the specific surface area with a maximum increase of 38% for SiMGF samples. Notably, the deviation between the experimental and FEA data was found to be below 11%. This shows that the FE model developed here provides reliable results comparable to experimental results. The trend reveals an increase in the specific surface area of the filler-to-matrix interphase by means of a porous structure, leading to an improvement in the mechanical properties at the interphase of GFRP composites. This improvement facilitated better physical interlocking and a smoother load transfer along the interphase. We also observed that an increase in specific surface area was necessary but not sufficient. For instance, GGF samples showed poor mechanical properties compared to their silica-coated counterparts with similar specific surface area (GGF compared to SimGF). This was ascribed to a lower adhesion of the graphene surface coating to the GF surface as a result of a lower chemical affinity.

The FE model reported here provides accurate technical information about the influence of the filler-to-matrix interphase structure on the mechanical properties of composites. This represents a reliable and low-cost alternative for the development of advanced composites by the rational design of its interphases. Furthermore, the model could also be implemented for studying other interphases in material science or as an input for more advanced simulations. This would allow us to address more sophisticated problems in composite technology, requiring high computational times.

## Figures and Tables

**Table 1:** Summary of the thickness, BET surface area (see Supplementary Materials), maximum load, experimental IFSS (Exp. IFSS), maximum shear stress and Mode II fracture for GFRP composites produced from: pristine (GF) and modified (SimGF, SiMGF, SiGGF and GGF) GFs. Parenthesis values represent the percentage increase in the experimental IFSS for modified GFs compared to pristine GFs. Data are plotted in Figure 3.

Type of Coating	Coating Thickness (nm)	BET (m^2^/g) [8]	Pull-Out Test		FEM Analysis	
			Max. Load (N)	Exp. IFSS (MPa)	Max Shear Stress (IFSS) (MPa)	Mode II Fracture (N/mm)
pristine GF	0	0.6	4.3 ± 0.1	9.8 ± 0.4	8.9	7.2
SimGF	100	4.8	4.7 ± 0.2	10.8 ± 0.5 (10%)	9.8	8.1
SiMGF	250	19.1	5.9 ± 0.1	13.6 ± 0.5 (38%)	12.3	10.5
SiGGF	256	10.7	5.0 ± 0.1	11.4 ± 0.6 (16%)	10.3	8.6
GGF	5000	0.85	4.6 ± 0.1	10.4 ± 0.6 (6%)	9.4	7.8

## Supplementary Materials

The following are available online at https://www.mdpi.com/article/10.3390/nano11102487/s1. Figure S1. SEM micrographs of SimGF, SiMGF, SiGGF and GGF. Figure S2. Scheme of the sample preparation and the pull-out test set up. Figure S3. Schematic view of three fracture modes along a crack. Figure S4. Force-displacement curves for pull-out tests carried out on all samples. Figure S5. SEM images of a bundle GF embedded into an epoxy drop and top view of an epoxy drop after pull-out test. Figure S6. Complete SEM characterization of the failure regions after pull-out test for GF, SimGF, SiMGF, SiGGF, and GGF composites.
