# Supplementary material for: A Finite Element Investigation into the Cohesive Properties of Glass-Fiber-Reinforced Polymers with Nanostructured Interphases
Source: Nanomaterials (Basel). 2021 Sep 24;11(10):2487. doi: 10.3390/nano11102487 (PMC8540472; doi:10.3390/nano11102487)
Supplement: Supplementary file 1 [file nanomaterials-11-02487-s001.zip › nanomaterials-1377474-supplementary.pdf]

Supplementary Materials

A Finite Element Investigation into the Cohesive Properties of Glass-Fiber-Reinforced Polymers with Nanostructured Interphases

Mohammad J. Ghasemi Parizi [1,2], Hossein Shahverdi [1,*], Ehsan Pipelzadeh [2,3], Andreu Cabot [2,4] and Pablo Guardia [2,5,*]

1. Aerospace Engineering Department and Center of Excellence in Computational Aerospace, AmirKabir University of Technology (Tehran Polytechnic), Tehran, 159163-4311, Iran; m.qasemi@aut.ac.ir
2. Catalonia Institute for Energy Research—IREC, Sant Adrià de Besòs, 08930, Spain; ehsanpipelzadeh@hotmail.com (E.P.); acabot@irec.cat (A.C.); pguardia@irec.cat (P.G.)
3. Chemical Engineering Department, University of Queensland, Brisbane, 4072, Australia
4. ICREA, Pg. Lluís Companys 23, Barcelona, 08010, Spain
5. Institute of Materials Science of Barcelona (ICMAB-CSIC), Campus UAB, Bellaterra, 08193, Spain

* Correspondence: h_shahverdi@aut.ac.ir (H.S.); pguardia@icmab.es (P.G.)

EXPERIMENTAL PROCEDURES

Preparation of wGF: A 2 x 30 cm^2 wGF fabric was immersed in a 1 M NaOH ethanol solution overnight. The wGFs were then sonicated in acetone, latter in ethanol and finally in MQ-water (10 min each). Finally, wGFs were dried in an oven at 50°C for 10 min.

Preparation of Silica thin film coatings: Silica thin films showing different structure were produced by following our recently reported approach (SimGF SiMGF and SiGGF) [1]. SimGF and SiMGF were produced by following a modified Stöber approach where suitable coatings allow for the production of a microporous or a mesoporous structures[2,3]. Samples were labeled SimGF- and SiMGF-wGF respectively (See Figure S1a-d). To grow an interconnected porous structure (gel) on top of the wGF, a sol-gel approach was modified [4]. The presence of an interconnected porous network would provide higher specific surface area and a better physical interlocking between the GF and the polymer matrix. Details of the process are published elsewhere [1]. The sample was labelled SiGGF-wGF (See Figure S1e-f).

Preparation of reduce graphene oxide (rGO)aerogel thin film coating: A rGO gel was grown on top of wGFs by performing a sequentially feeding gelating process in solution [5]. First, a 20 mL GO nanosheet feeding solution (0.25 mg/ml in MQ-water) was prepared by mixing a commercial solution of GO nanosheets (Graphenea S.L. 10 mg/mL) and MQ-Water in a 100 mL vial. Separately, a 20 mL solution containing the gelating/gelling agent was prepared by mixing ethylenediamine (EDA, sigma Aldrich, St. Louis, MO, USA) in water (1.5 mM final EDA concentration). Once the solutions were prepared, a 2 × 5 cm^2 of activated wGFs fabric was placed inside the feeding solution and aged in an oven at 80 °C for 1 h. Then, the fabric was removed, rinsed with MQ-water, and placed in the gelating/gelling solution. After aging the fabric for 1 h at 80 °C in an oven, this was removed and rinsed with MQ-Water. The fabric was placed again in the feeding solution and the cycle was repeated at least two times more. After three cycles, the fabric was placed in a petri dish, covered with water and placed in a fridge (−20 °C). Once the water was completely frozen, the petri dish was placed in a freeze dryer and left drying for two days (see Figure S1g,h).

Structural and physical characterization of modified GF: The surface morphology of modified wGF was characterized by low and high magnification scanning electron microscopy (SEM) using a Zeiss AURIGA operating at 4 kV. A piece of wGF fabric was placed onto carbon tape and coated with a copper thin film by sputtering to improve electrical conductivity. Specific surface area and porosity measurements were carried out by nitrogen physisorption at 77 K in a Micromeritics TriStar II machine. Prior to measurements, samples were degassed for 6 hours at 250 °C under nitrogen flow to eliminate weakly adsorbed molecules. The specific surface area was calculated using the multi-point Brunauer–Emmett–Teller (BET) model, considering 11 equally spaced points in the P/P^0 range from 0.05 to 0.30. The pore size distribution was evaluated from the desorption branches of isotherms according to the Barrett–Joyner–Halenda (BJH) method [6].

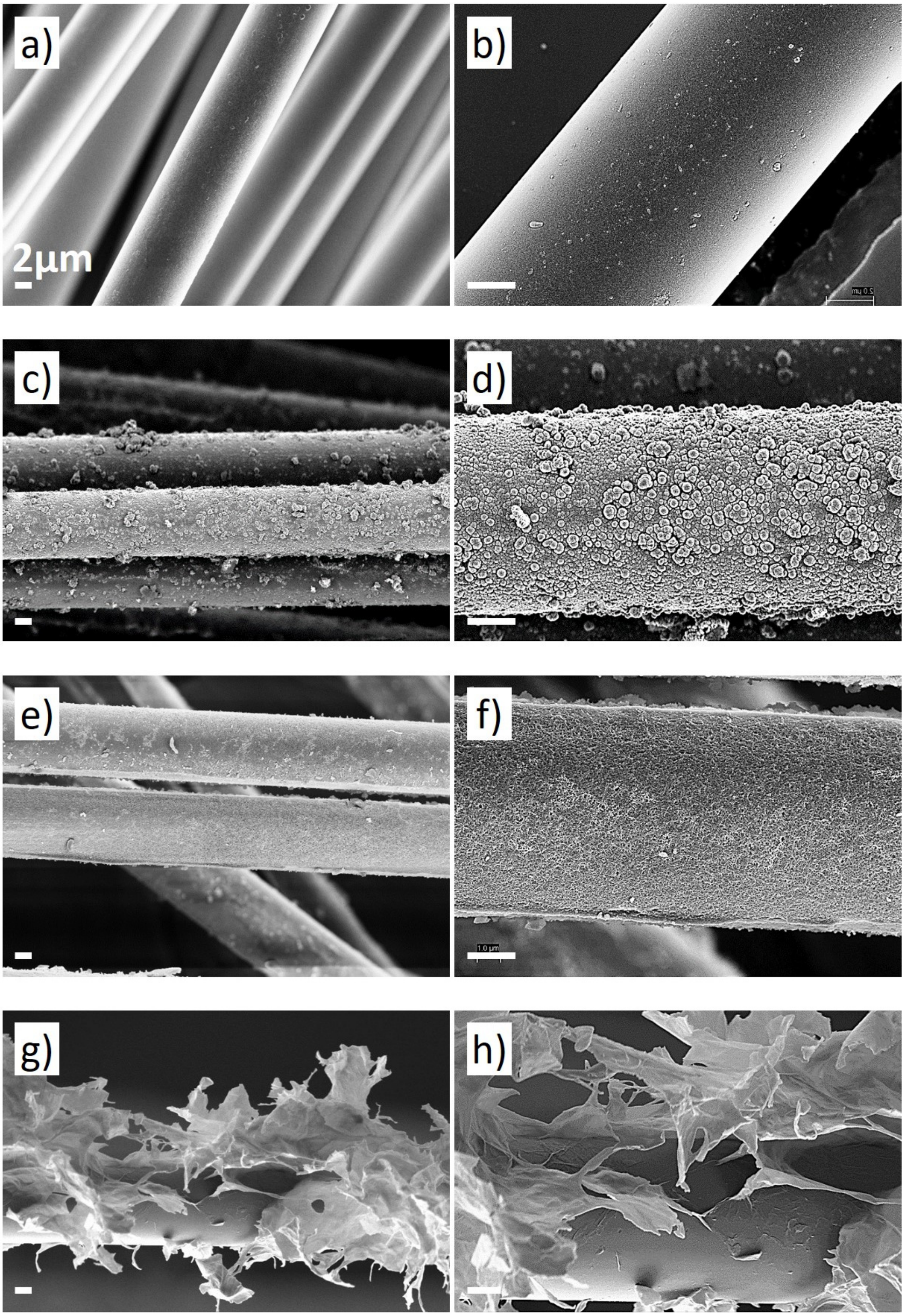

Figure S1. SEM micrographs of SimGF (**a, b**), SiMGF (**c, d**), SiGGF (**e, f**) and GGF (**g–h**). A part from SimGF, the thin film structure can be easily observed. For all samples, specific surface area values (Table 1) reveal an increase over the total surface area compare to pristine GF and hence the formation of a porous thin film coating. All scale bars represent 2 μm.

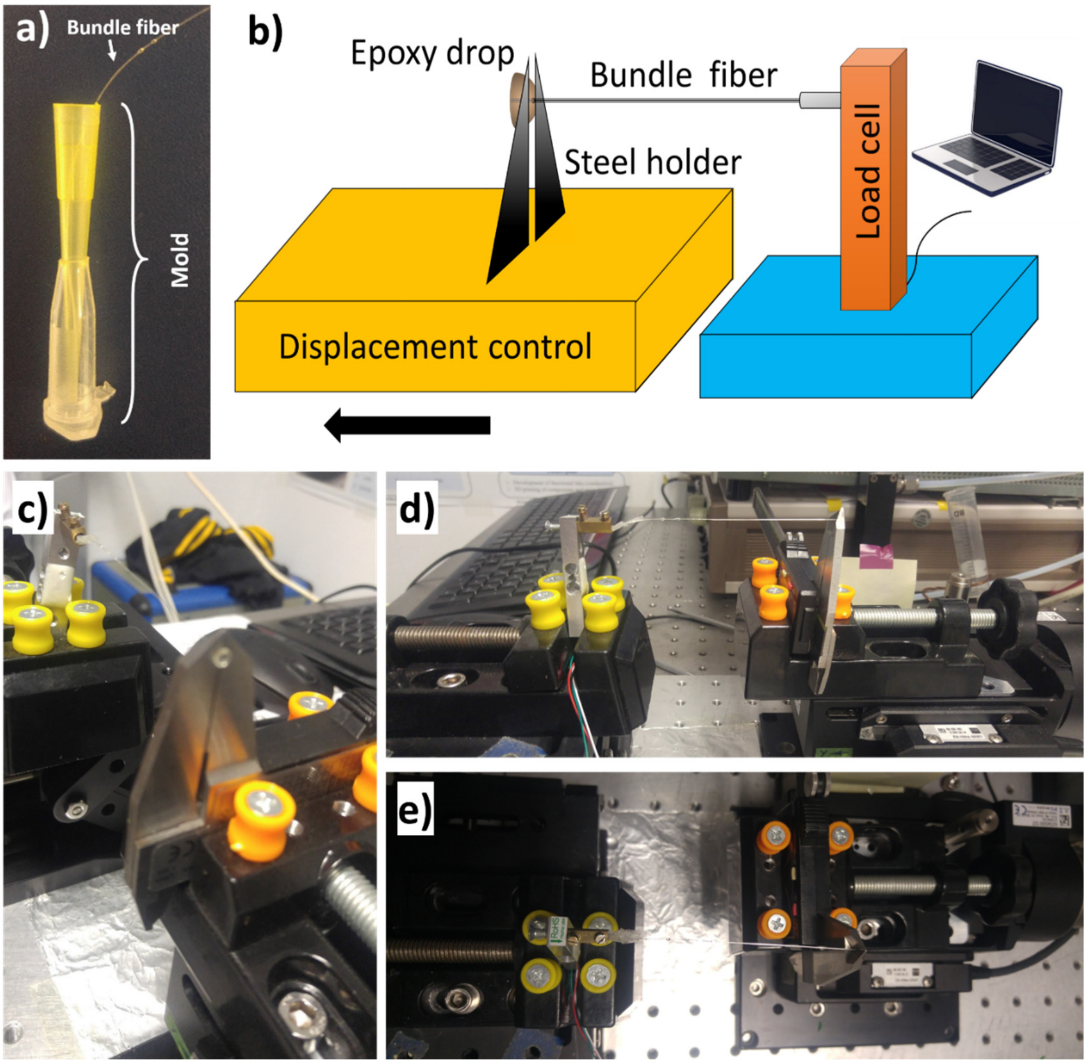

Figure S2. Scheme of (**a**) the sample preparation using a home-made sample holder and (**b**) the pull-out test set up. For the pull-out test, samples were placed in a steel holder (**c**) and the stage was moved in order to keep GF bundle parallel to the table before starting acquiring the displacement vs voltage curve. Side (**d**) and top (**e**) views of the experimental setup for pull-out test. Note that the load cell is attached to a fixed platform while the extreme of the fiber bearing the epoxy drop is attached to a metal holder placed on a XY translation stage.

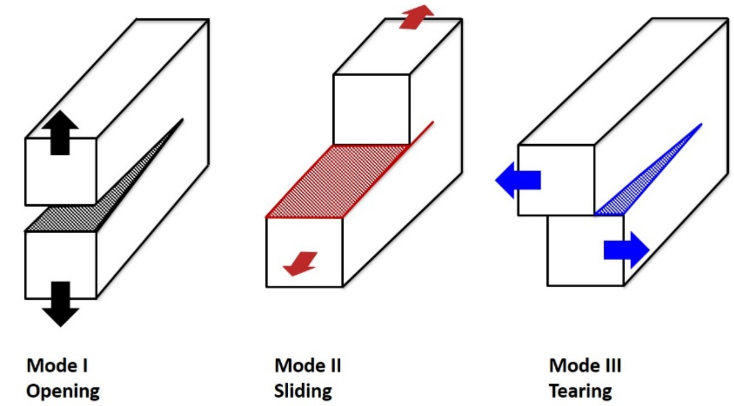

Figure S3. Schematic view of three fracture modes along a crack: Mode I is the opening mode, mode II is the shearing mode in the crack plane, and mode III is the tearing mode.

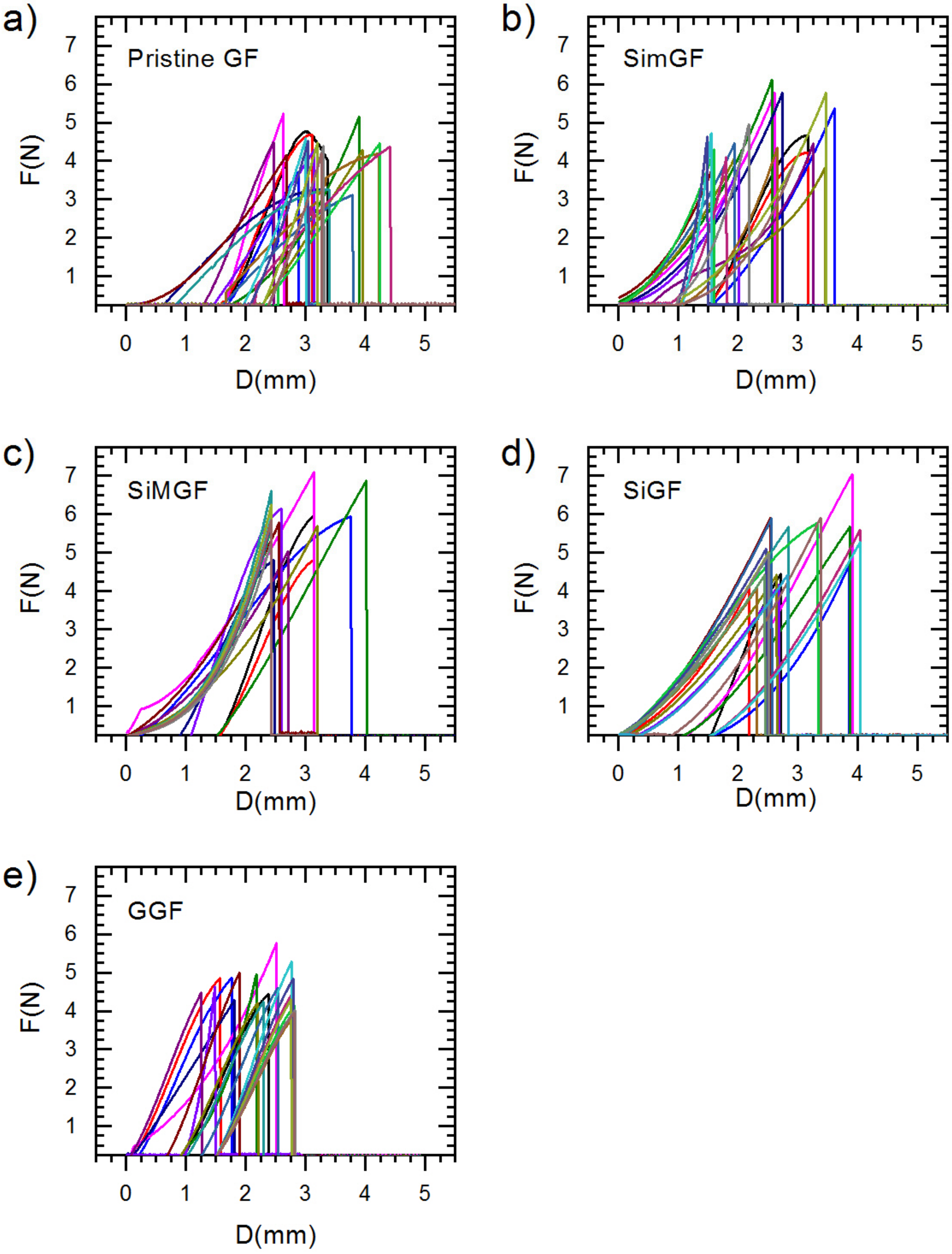

Figure S4. Force-displacement curves for pull-out tests carried out on all samples: (**a**) pristine GF, (**b**) SimGF, (**c**) SiMGF, (**d**) SiGGF, (**e**) GGF. Each test includes at least 15 measurements.

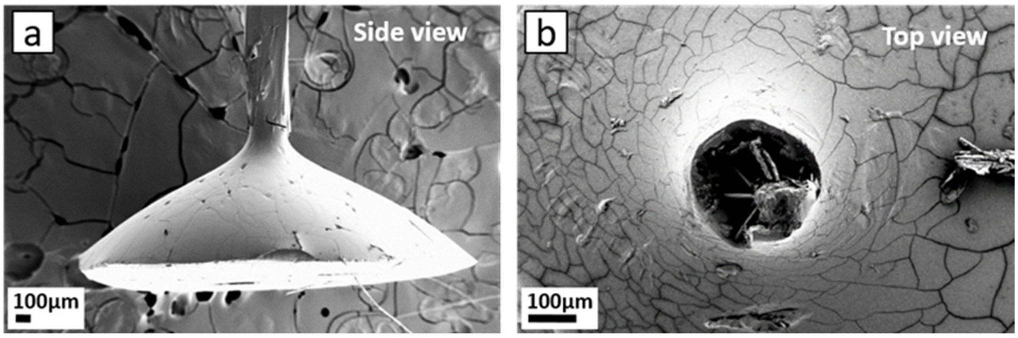

Figure S5. SEM images for the (a) side view of a bundle GF embedded into an epoxy drop and (b) top view of an epoxy drop after pull-out test.

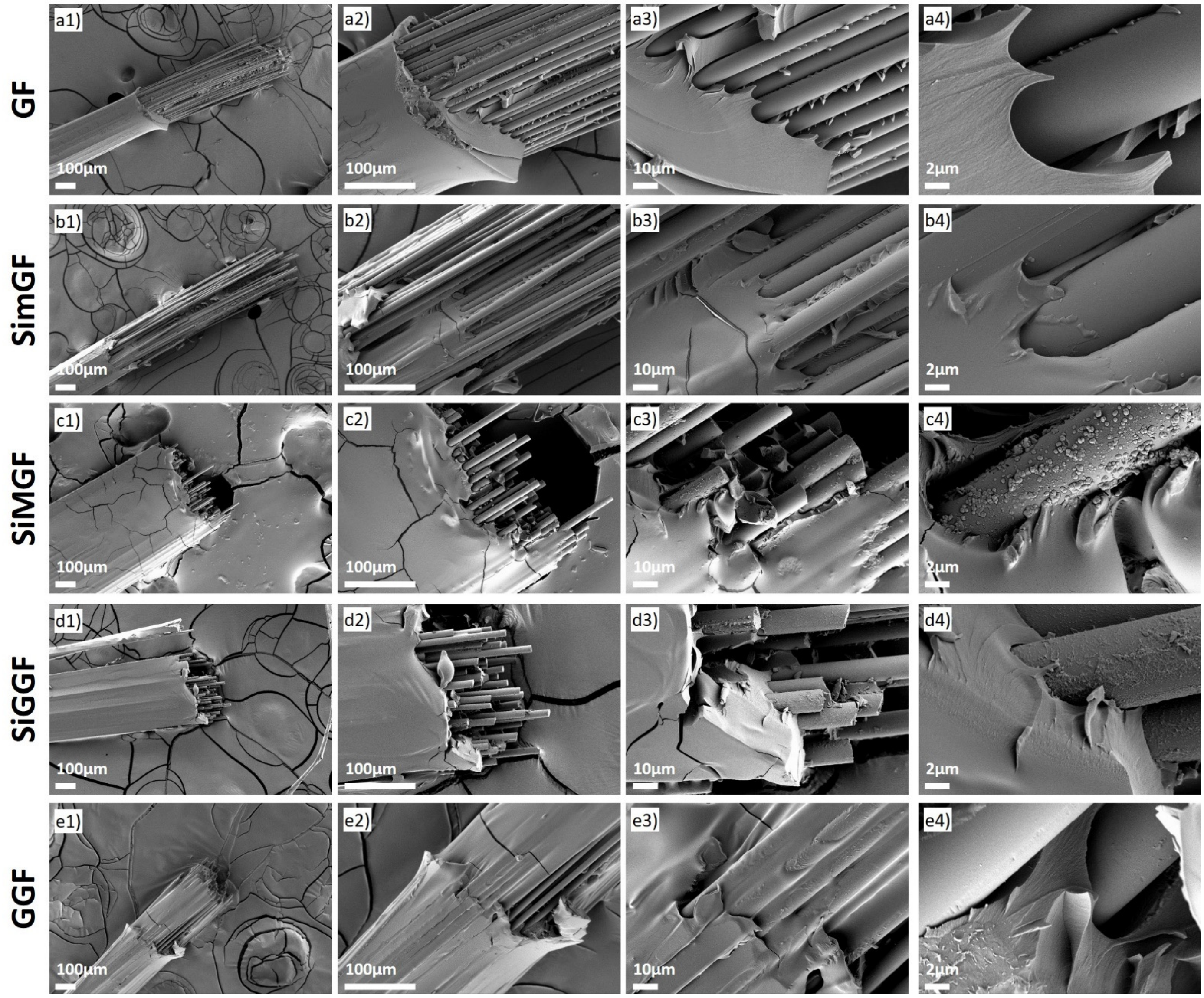

Figure S6. Complete SEM characterization of the failure regions after pull-out test for: (**a**) GF, (**b**) SimGF, (**c**) SiMGF, (**d**) SiGGF, and (**e**) GGF composites.

References

1. Parizi, M.J.G.; Shahverdi, H.; Roa, J.J.; Pipelzadeh, E.; Martinez, M.; Cabot, A.; Guardia, P. Improving Me-chanical Properties of Glass Fiber Reinforced Polymers through Silica-Based Surface Nanoengineering. *ACS Appl. Polym. Mater.* **2020**, *2*, 2667–2675, doi:10.1021/acsapm.0c00295.
2. Zhang, X.; Dou, Y.; Mou, C.-Y.; Asiri, A.M.; Teng, Z.; Li, W.; Zheng, G.; Zhao, D. Highly Ordered Mesoporous Silica Films with Perpendicular Mesochannels by a Simple Stöber-Solution Growth Approach. *Angew. Chemie Int. Ed.* **2012**, *51*, 2173–2177, doi:10.1002/anie.201108748.
3. Bao, Y.; Wang, T.; Kang, Q.; Shi, C.; Ma, J. Micelle-template synthesis of hollow silica spheres for improving water vapor permeability of waterborne polyurethane membrane. *Sci. Rep.* **2017**, *7*, 1–14, doi:10.1038/srep46638.
4. Nasir, T.; Herzog, G.; Hébrant, M.; Despas, C.; Liu, L.; Walcarius, A. Mesoporous Silica Thin Films for Improved Electrochemical Detection of Paraquat. *ACS Sensors* **2018**, *3*, 484–493, doi:10.1021/acssensors.7b00920.
5. Li, J.; Meng, H.; Xie, S.; Zhang, B.; Li, J.; Li, L.; Ma, H.; Zhang, J.; Yu, M. Ultra-light, compressible and fire-resistant graphene aerogel as a highly efficient and recyclable absorbent for organic liquids. *J. Mater. Chem. A.* **2014**, *2*, 2934–2941, doi:10.1039/c3ta14725h.
6. Thommes, M.; Kaneko, K.; Neimark, A. V.; Olivier, J.P.; Rodriguez-Reinoso, F.; Rouquerol, J.; Sing, K.S.W. Physisorption of gases, with special reference to the evaluation of surface area and pore size distribution (IUPAC Technical Report). *Pure Appl. Chem.* **2015**, *87*, 1051–1069, doi:10.1515/pac-2014-1117.
